# Assessment of Neonatal Circumcision Availability at Chicago-Area Hospitals

**DOI:** 10.1001/jamanetworkopen.2020.2306

**Published:** 2020-04-08

**Authors:** Ketan Jain-Poster, Ilina Rosoklija, Mateo Zambrano Navia, Max Maizels, Jane L. Holl, Matthew M. Davis, Emilie K. Johnson

**Affiliations:** 1 Northwestern University Feinberg School of Medicine, Chicago, Illinois; 2 Division of Urology, Ann & Robert H. Lurie Children’s Hospital of Chicago, Chicago, Illinois; 3 Department of Urology, Northwestern University Feinberg School of Medicine, Chicago, Illinois; 4 Department of Surgery, Northwestern University Feinberg School of Medicine, Chicago, Illinois; 5 Biological Sciences Division, University of Chicago, Chicago, Illinois; 6 Division of Academic General Pediatrics and Primary Care, Ann & Robert H. Lurie Children’s Hospital of Chicago, Chicago, Illinois; 7 Department of Pediatrics, Northwestern University Feinberg School of Medicine, Chicago, Illinois

## Abstract

This survey study assesses possible barriers to neonatal male circumcision by evaluating circumcision-related requirements and procedures among 44 birthing hospitals in the Chicago area in Illinois.

## Introduction

Our pediatric urology and surgery groups based in Chicago, Illinois, have noted clinically that young boys frequently present for delayed elective outpatient circumcision, often requiring general anesthesia. Although epidemiological and survey data indicate that neonatal circumcision rates are partly associated with health care resource availability,^1,2^ little is known to date about barriers to neonatal circumcision from clinician and hospital system perspectives. A prior survey found that 18% of parents seeking delayed circumcision for their son reported that, although they preferred immediate postnatal circumcision, their Chicago-area birthing hospital did not offer neonatal circumcision (9 hospitals represented).^3^ The present study seeks to describe neonatal circumcision-related requirements and procedures of birthing hospitals in the Chicago area to better understand barriers to neonatal circumcision experienced by clinicians and hospital systems.

## Methods

Publicly available data about each birthing hospital were combined with data from a brief, anonymous telephone survey conducted with a labor and delivery department representative at each hospital. Birthing hospitals (with at least 1 birth per year) within the Chicago area (Cook County, including the city of Chicago and adjacent suburbs) were identified on the Illinois Department of Public Health website.^4^ Characteristics including hospital type (government or nongovernment, not-for-profit status, and teaching status), location (city or suburbs), patient demographic characteristics (proportion publicly insured, race/ethnicity, and income by hospital zip code), and birth volume were collected.

Survey questions included whether neonatal circumcisions are performed at each hospital; whether they are performed for inpatients, outpatients, or both; and which specialty performs neonatal circumcisions. Representatives were asked whether insurance was accepted for neonatal circumcision or whether a separate cash fee was required. Descriptive statistics were calculated. Institutional review board determination from Ann & Robert H. Lurie Children’s Hospital of Chicago was for an exemption due to publicly available and anonymous data collection. A waiver of informed consent was granted.

## Results

In total, 44 birthing hospitals were identified (19 in the city [43%] and 25 in the suburbs [57%]; 38 not for profit [86%]; 11 teaching [25%]; median [interquartile range] births per year, 1413 [839.5-2224.5]) that serve patients from a wide range of socioeconomic levels and racial/ethnic groups (Table 1).

**Table 1.** Institutional Characteristics and Demographic Patterns of Patients at 44 Birthing Hospitals in the Chicago Area of Illinois

Characteristic	Data
Births/y, median (IQR), No.	1413 (839.5-2224.5)
Hospital location, No. (%)	
City	19 (43)
Suburb	25 (57)
Government hospital, No. (%)	
Yes	2 (5)
No	42 (95)
Teaching hospital, No. (%)	
Yes	11 (25)
No	33 (75)
Hospital for-profit status, No. (%)	
For profit	6 (14)
Not for profit	38 (86)
Publicly insured, median (IQR), %	66 (52-78)
Household income by hospital zip code, median (IQR), $	65 294 (41 110-84 556)
Racial/ethnic distribution by hospital zip code, median (IQR), %	
White	43.2 (17.3-75.5)
Black or African American	9.8 (3.1-56.7)
Asian	4.2 (0.1-7.1)
Hispanic or Latino	12 (6.2-24.4)
Other or >1 race	1.6 (0.1-2.2)

Abbreviation: IQR, interquartile range.

A representative responded from all 44 hospitals (100% response rate). All respondents reported offering neonatal circumcision (29 inpatient only [66%], 14 inpatient and outpatient [32%], and 1 outpatient only [2%]). Half reported accepting insurance for neonatal circumcision; the remainder were unsure whether insurance was accepted (vs requiring a cash fee). Obstetricians performed neonatal circumcision at 39 hospitals (89%) (Table 2).

**Table 2.** Specialties Performing Neonatal Circumcision in 44 Hospitals in the Chicago Area of Illinois

Specialty	No. (%)^a^
Obstetrics only	32 (73)
Obstetrics or family medicine	7 (16)
Urology	2 (5)
Other^b^	3 (7)

^a^ Percentages have been rounded and do not equal 100.

^b^ Includes family medicine or pediatrics (n = 1), pediatric surgery (n = 1), and general surgery (n = 1).

## Discussion

Birthing hospitals in the Chicago area serve patients from a range of socioeconomic levels and racial/ethnic backgrounds. In this study, all hospitals reported offering neonatal circumcision, although 18% of parents in a prior Chicago-area parent survey reported that their birthing hospital did not offer neonatal circumcision. The 5 hospitals most frequently represented in the prior survey^3^ had higher birth volumes (median, 2178 births/y), higher percentages of publicly insured patients (median, 78%), and higher percentages of black or African American patients (median, 26%) compared with all birthing hospitals in the present study.

The universally reported availability of circumcision contrasts with our patients’ experiences and with other studies that suggest that neonatal circumcisions are not always available.^5^ Only half of hospitals in the present study could confirm acceptance of insurance for neonatal circumcision (vs requiring cash payment), despite the American Academy of Pediatrics’ recommendation of providing coverage for all desired neonatal circumcisions.^6^ The present study’s findings indicate that publicly funded neonatal circumcisions may not be adequately incentivized, providing a target for future research and health policy initiatives.

This study is limited by surveying only 1 representative from each hospital, who may have had limited knowledge of institutional circumcision practices and policies. Also, reasons for the discrepancy between a prior parent survey and reported universal neonatal circumcision availability are unclear. The present study data may guide purposeful sampling of clinicians and administrators for future in-depth interviews, which can address these limitations. Interviews should seek to elucidate institutional best practices for, and barriers to, providing neonatal circumcision for all families who desire the procedure.
